# Increased pulmonary arteriolar tone associated with lung oxidative stress and nitric oxide in a mouse model of Alzheimer's disease

**DOI:** 10.14814/phy2.12953

**Published:** 2016-09-07

**Authors:** Andrew M. Roberts, Rekha Jagadapillai, Radhika A. Vaishnav, Robert P. Friedland, Robert Drinovac, Xingyu Lin, Evelyne Gozal

**Affiliations:** ^1^ Department of Physiology School of Medicine University of Louisville Louisville Kentucky 40202; ^2^ Department of Pediatrics School of Medicine University of Louisville Louisville Kentucky 40202; ^3^ Department of Neurology School of Medicine University of Louisville Louisville Kentucky 40202; ^4^ Department of Anatomical Sciences and Neurobiology School of Medicine University of Louisville Louisville Kentucky 40202; ^5^ Department of Thoracic Surgery the First Hospital of Jilin University Changchun China

**Keywords:** Amyloid precursor protein, endothelial dysfunction, lung microcirculation, neuroinflammation, reactive nitrogen species

## Abstract

Vascular dysfunction and decreased cerebral blood flow are linked to Alzheimer's disease (AD). Loss of endothelial nitric oxide (NO) and oxidative stress in human cerebrovascular endothelium increase expression of amyloid precursor protein (APP) and enhance production of the A*β* peptide, suggesting that loss of endothelial NO contributes to AD pathology. We hypothesize that decreased systemic NO bioavailability in AD may also impact lung microcirculation and induce pulmonary endothelial dysfunction. The acute effect of NO synthase (NOS) inhibition on pulmonary arteriolar tone was assessed in a transgenic mouse model (TgAD) of AD (C57BL/6‐Tg(Thy1‐APPSwDutIowa)BWevn/Mmjax) and age‐matched wild‐type controls (C57BL/6J). Arteriolar diameters were measured before and after the administration of the NOS inhibitor, L‐NAME. Lung superoxide formation (DHE) and formation of nitrotyrosine (3‐NT) were assessed as indicators of oxidative stress, inducible NOS (iNOS) and tumor necrosis factor alpha (TNF‐*α*) expression as indicators of inflammation. Administration of L‐NAME caused either significant pulmonary arteriolar constriction or no change from baseline tone in wild‐type (WT) mice, and significant arteriolar dilation in TgAD mice. DHE, 3‐NT, TNF‐*α*, and iNOS expression were higher in TgAD lung tissue, compared to WT mice. These data suggest L‐NAME could induce increased pulmonary arteriolar tone in WT mice from loss of bioavailable NO. In contrast, NOS inhibition with L‐NAME had a vasodilator effect in TgAD mice, potentially caused by decreased reactive nitrogen species formation, while significant oxidative stress and inflammation were present. We conclude that AD may increase pulmonary microvascular tone as a result of loss of bioavailable NO and increased oxidative stress. Our findings suggest that AD may have systemic microvascular implications beyond central neural control mechanisms.

## Introduction

Alzheimer's disease (AD) characteristically involves progression of amyloid‐*β* (A*β*) pathology in the brain and is a multifactorial process associated with oxidative stress, inflammation, and neurovascular damage. A*β*, derived from amyloid precursor protein (APP), causes neuroinflammation that leads to microvascular injury, dysfunction, and neurodegeneration (Sutton et al. 1999; Dudal et al. 2004; Paul et al. 2007). Thus, in the brain, AD has intertwined vascular and neurodegenerative components (De la Torre 2004). Cerebral amyloid angiopathy is a hallmark of AD (Thomas et al. 1996; Sutton et al. 1997) and increased A*β* is recognized as a cause of vascular endothelial cell damage, causing microvascular dysfunction via nitric oxide (NO) signaling (Thomas et al. 1996; Sutton et al. 1997) and susceptibility to ischemic brain damage (Zhang et al. 1997). An inflammatory response is a major initiating factor in AD with regard to both endothelial and neuroinflammation (Langer and Chavakis 2013).

Microcirculatory deficiency as an initiator of neurodegeneration in AD is supported by evidence of altered brain capillary structure, changes in endothelial shape, and impaired NO synthesis that affect signaling pathways common to the cardiovascular and nervous systems (De la Torre and Stefano 2000). A general finding is that the basal level of NO synthesis changes in many tissues, thereby affecting signaling molecules having inhibitory or excitatory cellular actions. Loss of constitutive NO impairs vasodilation of cerebral blood vessels, abolishes its inhibitory effect on platelet and leukocyte–endothelial adhesion, and increases inflammatory signaling, oxidative stress, and expression of adhesion factors and proinflammatory cytokines (Huang et al. 1994; DeCatarina et al. 1995; Lee 2000). The presence of A*β* promotes a loss of constitutive NO in the brain contributing to a proinflammatory state with hemodynamic disturbances, altered signaling pathways, and excessive inducible NO expression that promote vascular and neural injury (DeCatarina et al. 1995; Thomas et al. 1996; Sutton et al. 1997; De la Torre and Stefano 2000). Decreased microvascular blood flow in the brain causes an energy crisis that affects brain cells and their environment (De la Torre 2004). It is widely acknowledged that a variety of cerebral and systemic cardiovascular disturbances that cause cerebral hypoperfusion are initiating factors in the progressive neurodegenerative injury process of AD. As oxidative stress increases, bioavailable NO is depleted and excessive NO production via inducible nitric oxide synthase (iNOS) reacts with superoxide to form harmful reactive nitrogen species such as peroxynitrite (Ischiropoulos 1998).

Cardiorespiratory abnormalities such as sleep‐disordered breathing (Kadotani et al. 2001) and hypertension (Skoog and Gustafson 2006) have been associated with AD and may be related to the neuropathology of the AD process. However, there is a possibility that the inflammatory process and vascular injury could be extended to the systemic circulation and promote cardiorespiratory abnormalities. In the lungs, NO has a significant role in regulating microvascular blood flow, and loss of a basal level of endothelial NO synthesis may lead to an endotheliopathy that results in microvascular dysfunction and reduced perfusion (Sedoris et al. 2009). Acute lung injury, such as that caused by ischemia–reperfusion injury or inflammation, causes arteriolar constriction, platelet–endothelial adhesion, and increases pulmonary vascular resistance which can act to decrease alveolar blood flow and the ventilation/perfusion ratio (Miller and Roberts 1999; Ovechkin et al. 2005; Sedoris et al. 2011). If the AD inflammatory process reaches the lungs, as it may have the potential to do via the circulatory system (Ravi et al. 2004), we speculated that the lungs would undergo a loss of basal release of constitutive NO. Less bioavailable NO, with overproduction of NO via iNOS that reacts in the presence of oxidative stress to form harmful reactive oxygen and nitrogen species, would injure the endothelium, altering pulmonary microvascular responsiveness.

We hypothesized that the AD pathology is not limited to the brain and has systemic effects resulting from a systemic inflammatory process that would affect NO regulation of pulmonary microvascular tone. This possibility was examined in the present investigation by measuring microvascular changes, formation of 3‐nitrotyrosine (3‐NT), oxidative stress, and inflammation in the intact ventilated lungs of a transgenic model of AD in mice (TgAD). Consequences of decreasing lung NO bioavailability were examined by determining effects of acute NO synthase (NOS) inhibition with L‐NAME on pulmonary arteriolar baseline tone (arteriolar diameter) in the area of gas exchange in the right lungs of anesthetized, open chest TgAD and age‐matched wild‐type (WT) mice by intravital microscopy. Superoxide and 3‐NT formation, and iNOS and tumor necrosis factor alpha (TNF‐*α*) expression, were measured in lung tissue to assess changes in NO bioavailability and inflammation in conjunction with microvascular responses. Evidence of altered pulmonary microvascular responses, inflammation, formation of 3‐NT, and increased oxidative stress in the lung could indicate that the AD process initiates pulmonary microvascular dysfunction through a systemic inflammatory effect, potentially compromising lung function.

## Materials and Methods

### Surgical procedure

This protocol was approved by the University of Louisville Institutional Animal Care and Use Committee in compliance with United States Public Health Service standards and National Institutes of Health guidelines. It is in compliance with federal laws and regulations and the Guiding Principles in the Care and Use of Vertebrate Animals, published by the American Physiological Society. Experiments were done using a transgenic mouse model of Alzheimer's disease (C57BL/6‐Tg(Thy1‐APPSwDutIowa)BWevn/Mmjax) which expresses the human amyloid beta precursor protein (APP gene, 770 isoform) with the Swedish K670N/M671L, Dutch E693Q, and Iowa D694N mutations under the control of the mouse thymus cell antigen 1, theta, thy 1 promotor. These transgenic mice (TgAD) and wild‐type (WT) age‐matched male mice (C57BL/6J; BALB/C), which served as controls, were obtained from Jackson Laboratories (Bar Harbor, ME). Mice were anesthetized intraperitoneally with pentobarbital sodium (60 mg/kg). Supplemental doses of anesthesia (~7 to 15 mg/kg i.p.) were given as needed to maintain an adequate level of anesthesia as defined by the absence of whisker twitching, and a withdrawal reflex to a toe and tail pinch. The trachea was cannulated with PE 160 tubing, narrowed at one end to fit within the trachea, and inserted below the larynx. The mice breathed room air spontaneously and the left carotid artery was cannulated, with PE 10 tubing, to record arterial blood pressure with a Statham P23ID pressure transducer and a Grass model 7 polygraph recording system (Quincy, MA). After conclusion of the protocols described below, the mice were euthanized by an overdose of sodium pentobarbital anesthetic (approximately ≥ 60 mg/kg) followed by exsanguination. Lungs were removed and snap frozen in liquid nitrogen before being stored in a freezer at −80°C for subsequent analysis.

### Microvascular observations

Mice were placed on a temperature‐controlled heating pad on the stage of a modified trinocular microscope (Zeiss Axiolab, Thornwood, NY) equipped with epi‐illumination. The tracheal cannula was connected to a volume‐cycled ventilator (CWE model CTP‐930) and the lungs were ventilated with air (V_*t*_: 0.15–0.2 mL and 30–40 breaths/min) according to standards for small mammals. After positioning a mouse on its left side, the chest was opened at the fourth and fifth intercostal space for observation of pulmonary arterioles as described previously (Ovechkin et al. 2005). The objective lens with a glass dipping cone was positioned above the right lung and lowered to the pleural surface for observations when the lungs were held inflated for brief periods (~1 to 2 min) by switching from the ventilator to a system that delivered O_2_ at constant pressure (~10 cm H_2_O). Between observations, a drop of normal saline was applied topically to the pleural surface to keep it moist as needed. A color video camera (Microimage video systems automaticam model A106) mounted on the microscope was used to transfer images to a high‐resolution color monitor (Sony model PVM‐1390) and to a video tape recorder (Sony Videocassette Recorder model SVO‐9600) to store the images for offline analysis. The combined optical and video magnification was approximately 550×. Images were subsequently digitized for analysis using an image analysis system (Image‐Pro Plus 7.0, Media Cybernetics, Rockville, MD) calibrated with a stage micrometer (Meiji Techno MA285, Tokyo, Japan) so that internal arteriolar diameters and segment lengths could be measured. Arterioles were identified by their branching pattern and flow distribution. In this study, a pulmonary arteriole, about 2–3 branching orders upstream from the alveolar capillaries, was selected for observation.

### Administration of L‐NAME

To inhibit NOS, 0.1 mL of a solution of L‐NAME (l‐N^G^‐nitroarginine methyl ester hydrochloride) (0.1 mg/g body weight) dissolved in 0.9% NaCl was slowly injected into the saline‐filled catheter in the left carotid artery. To clear the catheter (~0.2 mL dead space), 0.2 mL of heparinized saline (10 units/mL) was injected slowly over a period lasting approximately 1–2 min. Pulmonary arterioles were visualized, on average, 22 min after L‐NAME was given.

### Experimental protocols

#### Protocol 1

To determine baseline diameters of pulmonary arterioles in the alveolar region of the intact lung, we continually measured diameters of arterioles at intervals in six WT and six TgAD mice.

#### Protocol 2

To determine whether the microvascular response was altered by nonspecific inhibition of NOS, arteriolar diameters were measured at intervals following L‐NAME administration in six WT and six TgAD mice after they underwent protocol 1.

##### Immunohistochemistry and tissue staining

**Figure 1:**
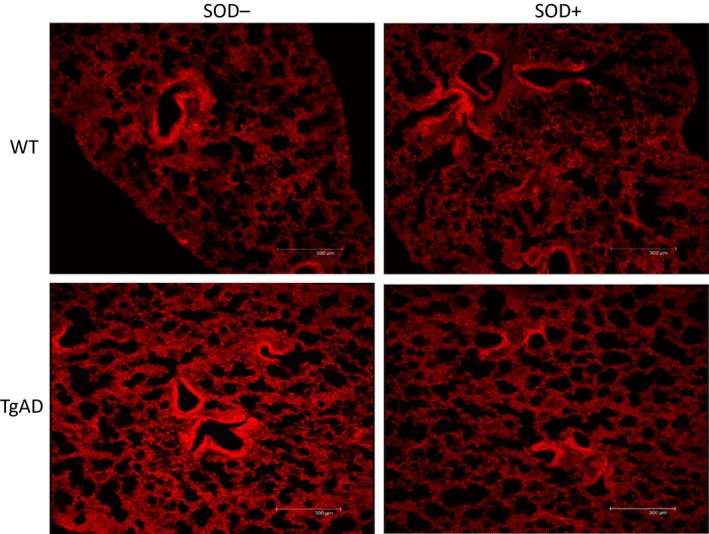
Comparison of DHE staining in lung tissue samples treated with (SOD+) or without superoxide dismutase (SOD−) in a wild‐type control (WT) and an Alzheimer's model mouse (TgAD). Note that fluorescence intensity decreased after SOD treatment in the TgAD mouse, while the lower levels in the WT mouse remained relatively constant.

Lung tissue was postfixed for 48 h in a 4% PFA solution. Following the fixation, the tissue was cryoprocessed in 15% sucrose and then 30% sucrose in PBS. Tissue was embedded in tissue freezing medium, cut into sections (30‐*μ*m thick), then mounted onto charged slides, and stained for superoxide by incubating 30 min in 20 *μ*mol/L dihydroethidium (DHE; Sigma Chemical Co, St Louis, MO). Specificity was confirmed by preincubating sections from the same mouse with 500 μ/mL superoxide dismutase (SOD; Sigma Chemical Co) for 30 min to ablate specific superoxide staining (Fig. 1). For immunofluorescence, sections were blocked with normal donkey serum and incubated overnight at 4°C with primary antibodies against iNOS (Abcam, Cambridge, MA) or 3‐NT (Millipore, Temecula, CA). The sections were incubated with FITC‐tagged anti‐rabbit secondary antibody (Jackson Immuno Research, West Grove, PA) for 1 h at room temperature in the dark. A negative control using normal isotype control IgG instead of primary antibody was used to control for nonspecific staining. Sections were then washed again with TBSTx, and mounted. Images were captured using a laser scanning confocal microscope (Leica‐TCS SL, Wetzlar, Germany). Images were then stored and analyzed by image analysis software (Image‐Pro Plus), and data were expressed as intensity per unit area.

##### Immunoblotting

Lungs from L‐NAME‐treated (*n* = 4 WT and *n* = 4 TgAD) and untreated animals (*n* = 4 WT and *n* = 4 TgAD) were homogenized in lysis buffer (0.4% NP‐40, 1% glycerol, 50 mmol/L tris at pH 7.5, 1 mmol/L Na orthovanadate, 150 mmol/L NaCl, 10 mmol/L EDTA, 100 mmol/L NaF, 20 mg/mL leupeptin, 10 mg/mL aprotinin, 0.5 mmol/L PMSF). Protein concentrations were measured using the BioRad Protein Assay Kit (BioRad, Temecula, CA). Equal amounts of proteins were separated on 10% Tris‐glycine gels (Invitrogen, Carlsbad, CA) and transferred onto nitrocellulose membranes for immunodetection with antibodies against iNOS and TNF‐*α* (Abcam, Cambridge, MA). Proteins were visualized with SuperSignal West Pico chemiluminescence (Thermo Scientific Pierce, Rockford, IL). Equal loading was verified by reprobing the membranes with *β*‐actin antibody (Sigma, St Louis, MO). Equal transfer of proteins onto nitrocellulose membranes was verified by Ponceau‐S staining. Immunoblots of lung lysates were repeated at least three times.

### Measurements and statistical analysis

Recorded images were digitized onto a computer by using Image Pro Plus. Measurements were made using the image analysis software. Vessel diameter was expressed as the internal diameter of pulmonary arterioles, measured on average at 1‐sec intervals over approximately 30 sec periods from images recorded continuously during an observation period. For each arteriole, we chose a straight segment (about 40–60 *μ*m in length) and made three diameter measurements along the segment. The three measurements, averaged to give one measurement of internal diameter per second, were averaged over the length of the observation period. These average diameters were compared before (control) and after L‐NAME in WT and TgAD mice. Vessel data were normalized by expressing the change in vessel diameter as a percentage of the average value during the control period. Mean arterial blood pressure (MABP) was measured when the mice were placed on the microscope stage before and after the lungs were mechanically ventilated, and when they were ventilated and the chest was open. For each microvascular observation period, MABP was measured prior to switching from the ventilator, during constant lung inflation, and during microvascular observations with constant lung inflation.

DHE, 3‐NT formation, and iNOS expression were determined in three samples of right lung tissue from each mouse by analyzing specific fluorescence intensities with Image‐Pro Plus. Intensity thresholds for each of the three specific stains were determined automatically by using the analysis software's bright object identification. The total intensity in stained lung tissue samples was measured using the preset intensity thresholds that were determined using automatic bright object identification and kept constant. The lung tissue area was calculated by subtracting the dark background (i.e., alveolar spaces) determined by the Image‐Pro Plus automatic dark object identification, from the total image area. In each of the three tissue samples from a WT or TgAD mouse, the total fluorescence intensity was divided by the total lung tissue area and averaged to give a value for each mouse. The percent of total lung tissue area exhibiting fluorescence within the preset threshold was measured by dividing the signal area with the total tissue area and averaged for each tissue sample, as explained above. Results are reported as mean fluorescence intensity units (FIU) per total tissue area (*μ*m^2^). A Student's paired *t*‐test was used to compare differences between two responses within mice (before and after L‐NAME for arteriolar diameter and blood pressure) and a *t*‐test was used to compare data between the WT and TgAD groups. For immunoblotting data, one‐way analysis of variance (ANOVA) was followed by Tukey's multiple comparison tests analysis (using GraphPad Prism version 5.01 for Windows). Data are reported as mean values ± standard deviation. For all comparisons, differences were considered statistically significant at *P *≤* *0.05.

## Results

**Figure 2:**
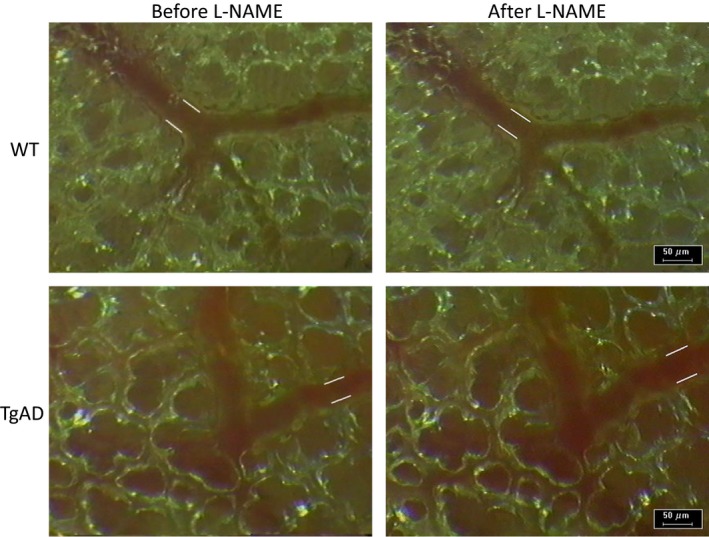
Intravital microscopic images of subpleural pulmonary arterioles comparing effects of a nitric oxide inhibitor (L‐NAME, 0.1 mg/kg body weight) on arteriolar diameter in a wild‐type (WT) control mouse and in a Alzheimer's disease model mouse (TgAD). White lines along vessels indicate interior arteriolar diameter. Note constriction in WT and dilation in TgAD.

**Figure 3:**
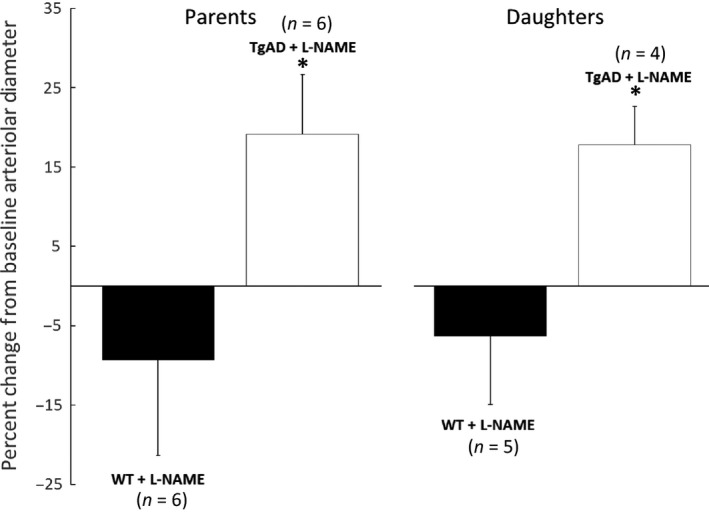
Comparison of the effect of L‐NAME on the change in parent and daughter branch pulmonary arteriolar diameters from their pre‐L‐NAME baselines in Alzheimer's model mice (TgAD, parent: *n* = 6) and wild‐type control mice (WT, parent: *n* = 6). Values are means ± SD. *Significant (*P* ≤ 0.05) difference between the responses of the TgAD and WT groups.

Subpleural pulmonary arterioles in the right lungs of WT (*n* = 6) and TgAD mice (*n* = 6) were examined before and after L‐NAME administration. The ages of the WT mice (26.7 ± 4.3 months, range: 20–31 months) were not significantly different from the TgAD mice (23.0 ± 3.2 months, range: 20–27 months). In addition, the body weights of the mice in the groups were similar (33.1 ± 1.2 g, range: 32.0–34.7 g vs. 31.7 ± 5.7 g, range: 26.8–39.0 g WT and TgAD, respectively). During baseline observation, before L‐NAME administration, the average diameter of the arterioles observed in WT (37.8 ± 10.3 *μ*m, range: 24.4–53.5 *μ*m) and TgAD mice (30.5 ± 6.9 *μ*m, range: 22.2–41.7 *μ*m) did not significantly differ. After L‐NAME treatment, the mean diameter from the WT mice decreased from baseline by 9.3 ± 12.0% (from 37.8 ± 10.3 *μ*m to 34.9 ± 11.7 *μ*m, range: 17.9–47.8 *μ*m), but this decrease did not reach significance. Of the six arterioles in the WT group, though three significantly increased their tone (−10.7%, −20.5%, and −26.6%), three did not significantly change their tone (0.2%, 0.3%, and 1.5%). In contrast, after L‐NAME, TgAD mean arteriolar diameter increased significantly compared to baseline by 19.2 ± 7.5% (from 30.5 ± 6.9 *μ*m to 36.1 ± 7.1 *μ*m, range: 27.8–46.4 *μ*m). The tone of all six arterioles in the TgAD group significantly decreased on average by 19.2 ± 5.5% (11.3%, 13.2%, 13.9%, 20.5%, 27.9%, and 28.1%). The increase in arteriolar diameter in the TgAD mice after L‐NAME treatment was significantly greater than the decrease in arteriolar diameter in the WT group (Figs. 2 and 3).

In five of the six WT and in four of the six TgAD, we observed branches of the initial arteriole. Average baseline diameters of the branches in WT (28.0 ± 6.4 *μ*m, range: 22.0–39.0 *μ*m) and TgAD (26.4 ± 1.4 *μ*m, range: 24.7–28.0 *μ*m) were not significantly different. However, after L‐NAME treatment, mean diameter of the arteriolar branches from the WT mice decreased by 6.3 ± 8.6% (from 28.0 ± 6.4 *μ*m to 26.4 ± 7.5 *μ*m, range: 20.7–39.2 *μ*m) compared to baseline, but this decrease did not reach significance. Of the five branches in the WT group, two increased their tone (−8.6% and −20.4%) and three had minimal changes (−1.8%, −1.1%, and 0.5%). In contrast, TgAD arteriolar diameter increased significantly from baseline after L‐NAME by 17.8 ± 4.9% (from 26.4 ± 1.4 *μ*m to 31.0 ± 1.0 *μ*m, range: 29.9–32.0 *μ*m). The tone of all four branches in the TgAD group decreased (13.0%, 14.3%, 21.1%, and 22.9%). In both groups, branches responded similarly to their parent arterioles and the increase in arteriolar diameter in TgAD after L‐NAME treatment was significantly (*P* ≤ 0.05) greater than the decrease in arteriolar diameter in WT (Fig. 3).

**Figure 4:**
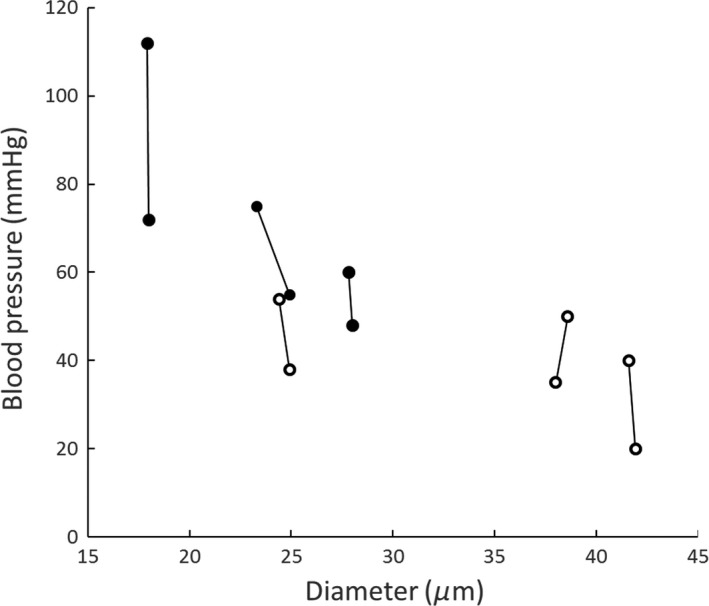
Pulmonary arteriolar diameter at different mean arterial blood pressures with (black circles) or without (open circles) the presence of L‐NAME. Each pair of diameter measurements is within a single mouse (*n* = 6).

When anesthetized mice were placed on the stage of the intravital microscope and were breathing spontaneously, MABP was not significantly different in the two groups (Table 1). After the lungs were mechanically ventilated and mice were placed on their left side, although MABP generally decreased, it decreased similarly in both groups (Table 1). Administration of L‐NAME acutely increased MABP similarly in WT and TgAD mice. There were no significant differences in MABP between the WT and TgAD mice when they were on the ventilator and the chest was opened, or during measurements prior to or after L‐NAME administration (Table 1). Furthermore, when the lungs were held briefly inflated at a constant pressure during control observations before L‐NAME, or during observations after L‐NAME, there were no significant differences in MABP between WT and TgAD mice (Table 1). Overall, microvascular diameter was fairly constant over a range of differences in MABP, either before or after L‐NAME administration (Fig. 4).

**Table 1:** Comparison of MABP in WT control and TgAD mice during different ventilatory conditions and during static lung inflation with microvascular observations

Condition	MABP	
Period	WT (mmHg)	TgAD (mmHg)
Spontaneous breathing	83.3 ± 11.3	81.2 ± 20.6
Mechanical ventilation	55.2 ± 13.5	60.0 ± 15.9
Mechanical ventilation + Open chest	45.8 ± 15.0	52.5 ± 15.0
Mechanical ventilation before constant inflation	50.5 ± 9.2	48.0 ± 18.8
Mechanical ventilation before L‐NAME	47.2 ± 10.9	47.3 ± 18.9
Mechanical ventilation after L‐NAME	84.3 ± 21.6^a^	87.3 ± 20.9^a^
Mechanical ventilation before constant inflation	70.2 ± 27.5	74.3 ± 24.8
Constant inflation: control observation	44.5 ± 14.2	42.7 ± 17.5
Constant inflation: L‐NAME observation	59.3 ± 30.3	55.0 ± 23.0

Values are means ± SD, *n* = 6 for each group.

MABP, mean arterial blood pressure, WT, wild type, TgAD, transgenic Alzheimer's disease model.

a *P* < 0.05 and denotes a significant difference in MABP after L‐NAME administration, compared to the baseline before L‐NAME. During each of the periods, there were no significant differences between the MABPs of the WT and TgAD.

**Figure 5:**
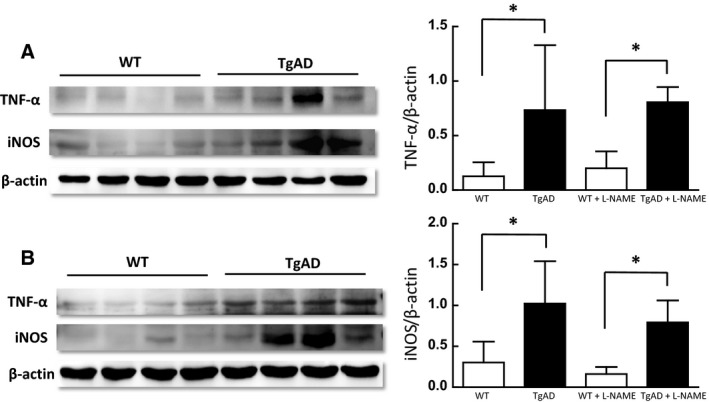
Left panel: Tumor necrosis factor alpha (TNF‐α) and inducible nitric oxide synthase (iNOS) immunoblotting in lung lysates from wild‐type (WT) and age‐matched transgenic Alzheimer's mice (TgAD) without (A) or with (B) L‐NAME treatment. Right panel: Densitometry quantification of TNF‐α (top panel) and iNOS (bottom panel) immunoreactivity normalized to *β*‐actin shows significantly increased TNF‐α and iNOS immunoreactivity in TgAD compared to WT mice (*P ≤ 0.05; *n* = 4). L‐NAME treatment did not significantly alter TNF‐α and iNOS immunoreactivity in any of the groups.

Immunoblotting of lung tissue quantified as the ratio of arbitrary density units normalized to *β*‐actin (Fig 5), showed increased expression of iNOS in TgAD mice without L‐NAME treatment (1.023 ± 0.517 TgAD vs. 0.301 ± 0.256 WT, *n* = 4, *P* ≤ 0.05) as well as in mice treated with L‐NAME (0.791 ± 0.269 TgAD vs. 0.160 ± 0.087 WT, *n* = 4, *P* ≤ 0.05). Similarly, without L‐NAME treatment, immunoblot analysis showed increased TNF‐*α* expression in TgAD mice compared to WT (0.734 ± 0.594 TgAD vs. 0.127 ± 0.128 WT, *n* = 4, *P* ≤ 0.05). In TgAD and WT mice treated with L‐NAME, TNF‐*α* expression was also greater in the TgAD mice (0.807 ± 0.138 TgAD vs. 0.200 ± 0.155 WT, *n* = 4, *P* ≤ 0.05). There were no significant differences in iNOS or TNF‐*α* expression between WT and TgAD mice with or without L‐NAME (Fig. 5).

**Figure 6:**
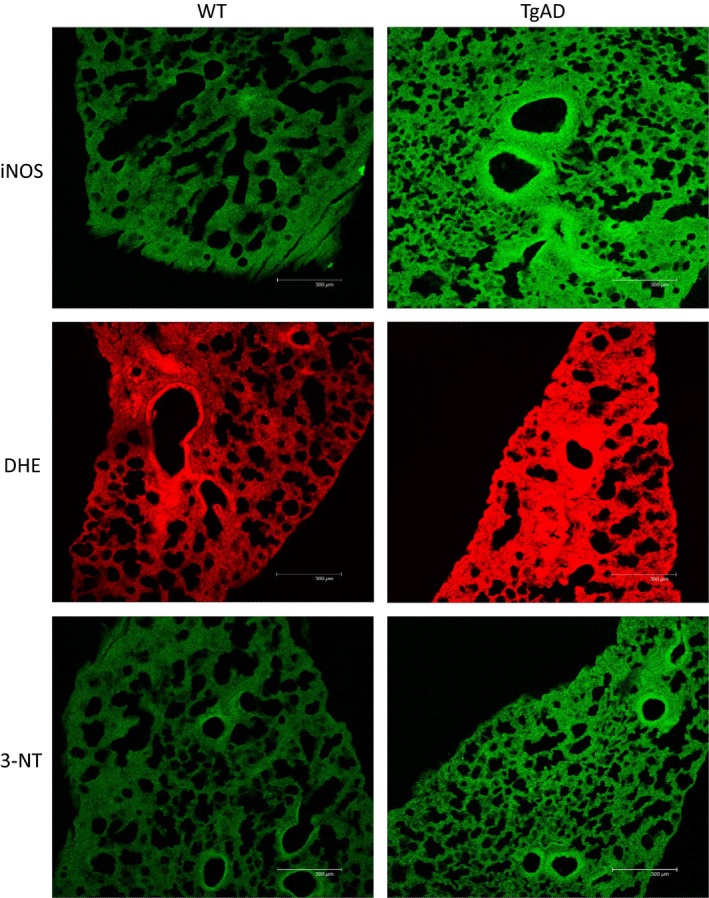
Comparison of inducible nitric oxide synthase (iNOS) expression, superoxide (DHE), and peroxynitrite (3‐NT) formation in lung tissue samples from a wild‐type control (WT) and an age‐matched (20 months) Alzheimer's disease model mouse (TgAD). Note increased fluorescence intensity in the TgAD mouse compared to the WT mouse.

**Figure 7:**
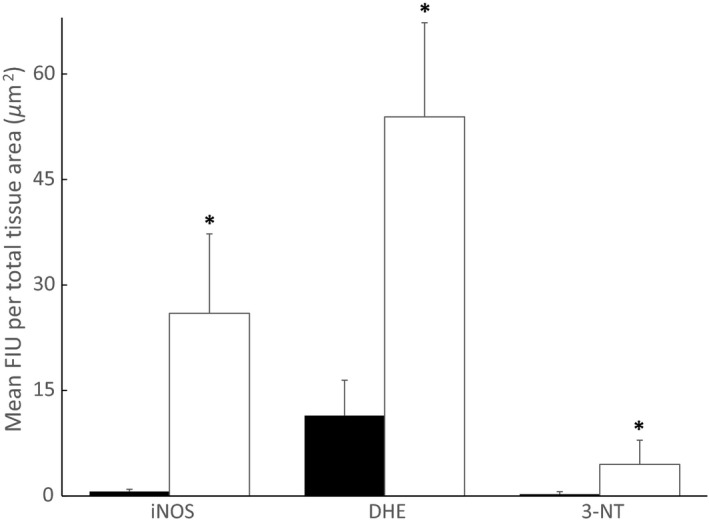
Comparison of mean fluorescence intensity units (FIU) per total tissue sample area for inducible nitric oxide synthase (iNOS) expression, superoxide (DHE), and peroxynitrite (3‐NT) production in Alzheimer's model (TgAD, *n* = 4, open bars) and wild‐type (WT, *n* =4, black bars) mice. Values are means ± SD. *Significant (*P* ≤ 0.05) difference between WT and TgAD.

**Figure 8:**
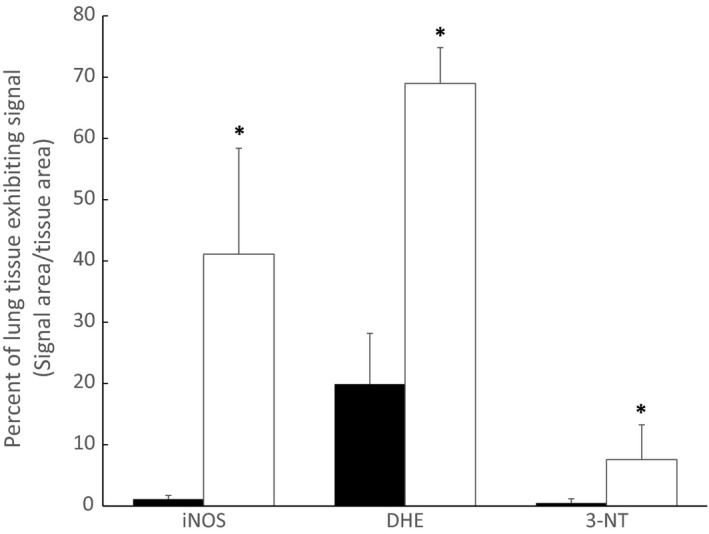
Comparison of percent of lung tissue exhibiting standard intensity for inducible nitric oxide synthase (iNOS) expression, superoxide (DHE), and peroxynitrite (3‐NT) production in Alzheimer's model (TgAD, *n* = 4, open bars) and wild‐type (WT, *n* = 4, black bars) mice. Values are means ± SD. *Significant (*P* ≤ 0.05) difference between WT and TgAD.

Immunohistochemical analysis of iNOS expression and 3‐NT formation, as well as staining for superoxide production (DHE), showed significantly greater mean FIUs per tissue area in TgAD mice (26.0 ± 11.3 FIU/*μ*m^2^, 4.5 ± 3.4 FIU/*μ*m^2^, 53.9 ± 13.4 FIU/*μ*m^2^, respectively, n = 4) than in WT controls (0.7 ± 0.3 FIU/*μ*m^2^, 0.3 ± 0.4 FIU/*μ*m^2^, 11.4 ± 5.0 FIU/*μ*m^2^, respectively, n = 4) (Figs 6 and 7). In addition to greater fluorescence intensity in the TgAD mice, the percent of lung tissue area exhibiting immunofluorescent staining for iNOS expression, 3‐NT formation, and DHE production was significantly greater in TgAD (41.1 ± 17.3%, 7.6 ± 5.7%, 69.0 ± 5.8%, respectively, *n* = 4) compared to WT (1.1 ± 0.6%, 0.5 ± 0.7%, 19.9 ± 8.3%, respectively, *n* = 4) (Fig. 8).

## Discussion

In this study, we investigated whether the AD pathogenic process causes pulmonary microvascular dysfunction associated with altered NO expression and oxidative injury in the lungs of AD transgenic mice, compared to their age‐matched wild‐type controls. Based on the well‐described cerebral microvascular injury and dysfunction initiated by inflammatory effects of A*β* and altered NO synthesis, identification of a similar injury in the pulmonary microcirculation would indicate that AD may have systemic microvascular effects in vulnerable tissues.

Our results showed that acute administration of L‐NAME to inhibit total NOS dilated pulmonary arterioles in TgAD mice and decreased their basal tone. In contrast, L‐NAME caused constriction of pulmonary arterioles of age‐matched WT controls or did not cause a significant change in their diameter. The variable increase in arteriolar tone in response to L‐NAME in aged WT mice could be due to the incidence of decreased NO bioavailability in aged WT C57BL/6J mice as indicated by others (Yang et al. 2009). Together, these findings suggest that NO availability is altered in pulmonary arterioles in the lungs of TgAD mice and imply that basal pulmonary arterial tone is elevated by the AD process. This result is similar to what has been found regarding microvascular effects of AD in brain and retinal microvessels concerning endothelial effects on vascular regulatory mechanisms (Lee 2000; Iadecola 2004; Cheung et al. 2014) and agrees with evidence that TgAD mice have impaired responses to endothelium‐dependent vasodilators related to loss of basal NO availability and upregulation of superoxide dismutase (Tong et al. 2005).

Furthermore, comparison of lung samples from the TgAD and WT mice showed significantly elevated iNOS expression, superoxide production, and 3‐NT generation in the TgAD group compared to the WT group. These findings are analogous to previous reports of the effects of A*β* in brain tissue where alterations in NO signaling have a central role in causing microvascular dysfunction in AD (Gentile et al. 2004). Increased TNF‐*α* expression in the TgAD mice could likely contribute to the microvascular abnormality in the TgAD group through inflammatory and oxidative processes as previously described in brain tissue with AD. Interestingly, these findings are similar to what we and others reported for microvascular dysfunction and NO signaling with acute lung injury such as that caused by ischemia–reperfusion (Sedoris et al. 2009). Like in the brain, basal constitutive NO bioavailability and signaling in the lung are major regulating factors in the pulmonary circulation.

AD is linked with brain oxidative stress and inflammation (Hamdheydari et al. 2003) and has been associated with cardiopulmonary disorders. We believe that the current study provides evidence that AD pathology is accompanied by pulmonary inflammation, oxidative injury, and microvascular dysfunction that may reduce bioavailability of NO in the pulmonary circulation. Changes in pulmonary vascular tone in AD may aggravate the physiological aging‐induced decrease in lung function, possibly as a result of increased superoxide formation and iNOS induction, causing further decreased bioavailable NO and increased protein nitrosylation. Superoxide reacts with NO with very high‐affinity forming peroxynitrite and as a result, posttranslational nitrosylation of protein, decreased NO bioavailability and endothelial injury (Ischiropoulos 1998). A limitation of the present study is that microvascular responses to administration of endothelium‐independent or ‐dependent vasodilators and constrictors were not tested. Future studies, beyond the scope of our initial experiments, are therefore needed to more directly assess vascular responsiveness to NO and to more directly measure NO and signaling pathways.

Our results show that while WT arterioles have the capacity to constrict in response to NOS inhibition with L‐NAME, this response is abrogated in TgAD mice, resulting in what appears to be a NO‐independent vasodilation. These data suggest that while NO combined with increased superoxide may maintain or increase vascular tone in AD mice, in the absence of NO when NOS is inhibited, AD‐induced lung oxidative stress results in loss of vascular tone and vasodilation. Additionally, NOS inhibition preventing peroxynitrite formation in the presence of superoxide could promote the formation of hydrogen peroxide (H_2_O_2_) from superoxide dismutation, a reaction catalyzed by antioxidant enzymes such as SODs. In vascular endothelial and smooth muscle cells, H_2_O_2_ has been identified as a signaling molecule promoting vasorelaxation (Graier 2008; Breton‐Romero and Lamas 2014). It has been shown in vivo that H_2_O_2_ mediates non‐NO and nonprostanoid‐dependent vasorelaxation to bradykinin, acting as an endothelium‐derived hyperpolarizing factor (EDHF) in the piglet pial vasculature, that was not blocked by L‐NAME, but was abrogated by catalase (Lacza et al. 2002). Several studies show that H_2_O_2_ acts like an EDHF and enhances EDHF formation that can compensate for the loss of available NO (Lacza et al. 2002; Thengchaisri and Kuo 2003; Graier 2008). Therefore, our data suggest that in contrast to WT where L‐NAME causes a loss of NO‐induced vasorelaxation resulting in vasoconstriction, TgAD arterioles may lack responsiveness to NO. Immunoblotting experiments show that acute NOS inhibition does not prevent iNOS and TNF‐*α* increases in TgAD mice lungs, maintaining the tissue inflammatory process, and only preventing NO release. Therefore, despite the persisting inflammatory environment, blocking the formation of NO in AD lungs does not result in vasoconstriction, but may only enhance vasodilation by increasing formation of H_2_O_2_.

The present results are not merely due to aging and are likely to result from multiple mechanisms that include: (1) loss of NO bioavailability, (2) oxidative stress, (3) production of hydrogen peroxide, and (4) altered production or responses to other vasoactive mediators, changing the balance between vasodilation and vasoconstriction. Further experiments, beyond the scope of the present study, are needed to define the mechanisms of altered basal arteriolar tone in the TgAD mice. Overall, our findings add a systemic component to the “vascular hypothesis” of AD (De la Torre 2004) and suggest that AD may have cardiopulmonary implications beyond neuroinflammation. Lung involvement in the AD process may potentially compromise gas exchange and lung function, resulting in tissue hypoxemia that could exacerbate the brain oxidative process and injury. The influence of the AD process on the lungs warrants further study to define mechanisms of pulmonary vascular dysfunction and potential implications for other peripheral vascular beds.

## Conflict of Interest

None declared.
